# The Contribution of Prefrontal Executive Processes to Creating a Sense of Self**

**DOI:** 10.4103/0973-1229.77432

**Published:** 2011

**Authors:** William Hirstein

**Affiliations:** * *Chair, Philosophy Department, Elmhurst College, Elmhurst, Illinois, USA.*; ** *Revised and peer reviewed version of a paper for an International Seminar on Mind, Brain, and Consciousness, Thane College Campus, Thane, India, January 13-15, 2010*

**Keywords:** *Consciousness*, *Executive processes*, *Self*, *Sense of self*

## Abstract

According to several current theories, executive processes help achieve various mental actions such as remembering, planning and decision-making, by executing cognitive operations on representations held in consciousness. I plan to argue that these executive processes are partly responsible for our sense of self, because of the way they produce the impression of an active, controlling presence in consciousness. If we examine what philosophers have said about the “ego” (Descartes), “the Self” (Locke and Hume), the “self of all selves” (William James), we will find that it fits what is now known about executive processes. Hume, for instance, famously argued that he could not detect the self in consciousness, and this would correspond to the claim (made by Crick and Koch, for instance) that we are not conscious of the executive processes themselves, but rather of their results.

## Introduction

The sense of the word “self” used in this paper’s title occurs most clearly in the work of philosophers John Locke and David Hume when they speak of a *Self*, either to assert its existence as Locke did, or to deny it, as Hume did. Rene Descartes is speaking about this sort of self when he uses the Latin term “ego.” William James also uses the word “self” in this sense, which I will call the *psychological sense*. This is the sense of “self” in which it is seen as an internal psychological entity, something at work in the mind, rather than the entire mind. Representations are present in our conscious states, but there is also a robust and enduring sense of something else at work there. As James said, “whatever content his thought may include, there is a spiritual something in him which seems to go out to meet these qualities and contents, whilst they seem to come in to be received by it” (James, 1890, p297-298). Sometimes thoughts and images just flow through our heads, but other times, we actively think: Representations are purposefully brought up from memory, compared with other representations and evaluated in other ways, and used to formulate plans of action. These are the sorts of mental events that give rise to the idea of a *psychological self*, something in the head summoning representations from memory, comparing them, accepting or rejecting them as real or as important, and finally, using them to plan and execute actions. This sort of self is not composed of representations, but rather it performs various functions on them.

More recently, the psychological self is showing signs of making a comeback in neuroscience, and specifically in neuropsychology. Crick and Koch say that a good heuristic for understanding the overall functional scheme of the brain’s cortex is “to imagine that the front of the brain is ’looking at’ the sensory systems, most of which are at the back of the brain” (Crick and Koch, 2003, p120). Baars and his co-authors endorse that quotation, after saying that “conscious experience in general can be viewed as information presented to prefrontal executive regions for interpretation, decision-making and voluntary control” (Baars *et al*., 2003, p673). They also noticed the self-like quality of executive processes, saying that they “can be viewed as properties of the subject, rather than the object, of experience–the ’observing self’” (Baars *et al*., 2003, p671).

My purpose here is to elucidate and argue for the claim that the psychological self is real and is embodied in a set of brain processes. If we list the functions of the psychological self as they are described by the classical philosophers, we can see that these functions correspond closely to the functions achieved by what neuroscientists call *executive processes*. The psychological self is embodied in the brain’s executive processes, according to this view. By showing that the list of executive functions corresponds well to the functions assigned to the psychological self, my hope is to ground this notion of self in existing theory of brain function. My aim is to show that, contrary to the sceptics, there is a perfectly good sense of “self”-nothing odd or esoteric-that applies straightforwardly to a set of brain processes. I am equating an old idea-that of the self-with a new one: the emerging neuroscientific theory of executive processes.

A second goal of this contribution is to show that we can make sense of a specific part of the philosophical debate about the self. Hume and James argued that we do not have direct conscious awareness of the self. This corresponds closely, I will argue, to what neuroscientists have said about executive processes: we are not directly aware of them, rather we are aware of the changes they affect in our conscious states. Hume complained that he was not able to sense any Self, rather just “some particular perception or other, of heat or cold, light or shade, love or hatred, pain or pleasure” (Hume 1739/1987, Book 1, section VI). Hume was not aware of a self, he was only aware of sensations associated with perception, emotions or feelings.

## Self *vs* Homunculus

Crick and Koch call their version of the psychological self “the unconscious homunculus,” using the phrase somewhat tongue-in-cheek, because they believe that our inability to be conscious of executive processes causes us to believe that they all are to be explained by the actions of a single entity-a homunculus-that has most of the mental abilities of a full-blown human being. They say that we “are not directly aware of inner world of thoughts, intentions, and planning (that is, of our unconscious homunculus), but only of the sensory representations associated with these mental activities” (Crick and Koch, 2000, p109). We are only aware of the representations that the executive processes call up (e.g., from memory) or produce (e.g., in the form of conscious, speech-like thoughts). According to them, “the unconscious homunculus receives information about the world through the senses, thinks, and plans and executes ’voluntary’ actions. What becomes conscious, then, is a representation of some of the activities of the unconscious homunculus in the form of imagery and spoken and unspoken speech” (Crick and Koch, 2000, p107).

Once we roughly equate the psychological self with the set of executive processes, we can see that the two following questions:

- Are we aware of the psychological self? and,
- Are we conscious of executive processes?

are fundamentally the same question. This equation also gives us insight into the homunculus fallacy. A homunculus can be seen as an implausible version of a psychological self, typically because the homunculus accomplishes all of the executive processes and in doing so seems to have all the mental capacity of a full person. What made people attribute all of the executive processes to a single psychological entity was their lack of direct awareness of those processes.

## The Self of the History of Philosophy is the Set of Executive Processes

The psychological self is at work in the mind, performing cognitive functions that Descartes groups together under the concept *thinking*:

But what then am I? A thing which thinks. What is a thing which thinks? It is a thing which doubts, understands, [conceives], affirms, denies, wills, refuses, which also imagines and feels (Descartes, 1967, Second Meditation).

Descartes is clear that by “feels,” he is referring to perception in general: “I am the same who feels, that is to say, who perceives certain things, as by the organs of sense, since it is true I see light, I hear noise, I feel heat.” This “I” he is describing sounds like the psychological self, and his list of functions it performs matches up quite well with a list of executive functions:

Am I not that being who now doubts nearly everything, who nevertheless understands certain things, who affirms that one only is true, who denies all the others, who desires to know more, is averse from being deceived, who imagines many things, sometimes indeed despite his will, and who perceives many likewise, as by the intervention of the bodily organs? (Descartes, 1967).

The psychological self encounters perceptual information as it enters through the sense organs. It is what James calls “the *active* element in consciousness” (James, 1890/1950, p297-298).

### Executive processes

In the back half of the brain, large multimodal representations of the world (as it is according to me) are assembled from input produced by each sensory modality. This information has itself already passed through many computational stages. These final multimodal representations are expensive to produce, update and maintain. It does not make sense to have representations if nothing is done with them. The primary reason for having representations of something is to use those representations in order to understand and affect that thing. There exist processes in the brain’s prefrontal lobes which perform different operations on our representations, the processes we commonly call by the collective name, thinking. Deciding, weighing, reasoning, inferring, examining, resolving, are all things we do with our mind/brains, but they do not happen out of nowhere, in some nonphysical medium, they happen somewhere in the brain. Executive processes, typically centred in the prefrontal lobes, perform functions on representations. As a unit, the prefrontal executive processes accept as input perceptions, memories or emotions, and produce motor activity in one of the motor systems as output. We know which neuron types tend to predominate at each of the cortical levels in the areas thought to house executive functions. We know which sensory modalities each prefrontal area receives–not all of them receive signals from all of the modalities. We also know which of the body’s effector systems each area sends signals to, including the eyes; the hands; the arms and legs; the mouth, tongue and throat and the autonomic system.

The first organisms to evolve and the simplest organisms existing today operate according to a very strict stimulus-response plan. They can detect a few things or properties out there, and respond with a few different behaviours. As organisms get more complex, they develop more and more of these inflexible perception-action cycles. But, because the environment’s true complexity is much richer than any reasonably sized set these cycles can account for, a new more powerful way of responding was developed, one with flexibility. Executive processes come into action when flexibility of response is needed. When we are engaged in well-practiced activities, such as driving home from work, washing the dishes, watching television and so on, the brain operates in a more automatic mode. But when something goes wrong, our normal route home is blocked or the television won’t come on, then we need to think, problem-solve, plan and execute more complex, less automatic behaviours, and this means that the executive processes have begun to operate.

Thus, executive processes are needed when there are no effective learned input-output links. When we attempt something new, such as learning how to play tennis, executive processes are required. If they are damaged, the person is simply unable to learn at a cognitive level (lower level behaviours can still be learned through a separate procedural memory system). As we get better at the new task, executive processes pass it to more posterior brain areas that specialise in efficiently performing routine actions without conscious interruption. A large body of brain imaging studies shows that as we become more practiced at something, the brain processes used switch from networks containing a heavy prefrontal component, to networks primarily residing in more central and posterior brain regions. Another general situation in which we use executive control occurs when there is some sort of danger. Executive control produces actions with the highest flexibility and the lowest probability of error. We react more slowly under executive control, but more effectively. Sometimes when a quick action is needed, there is no time for executive processes to work, and our actions are ineffective or unnecessary. If you have ever sat behind the backstop fence at a baseball game, you probably would have noticed that you cannot stop yourself from raising your hands and flinching when a foul ball heads directly at you, an unnecessary action because there was no time to correct it with executive processes.

Neuroscientists are currently exploring several different classification schemes for the executive processes, such as classification by function (Shallice, 2002; Baddeley, 2002) and classification by cortical areas they occupy (Stuss *et al*., 2002). Hence, in describing them, we need to make a fundamental choice between beginning with anatomy and moving to function, or vice versa. Fortunately, several factors converge here to make this decision easier: The exact nature of the functions is still very much up in the air, from theories according to which there are several specific functions, to theories that posit a few basic functions that are compounded repeatedly to achieve executive functions (Shallice, 2002). The basic anatomy, on the other hand, is clear, at least in terms of what cell types exist at which cortical levels, and how the cortical areas are connected.

## Executive Processes Interact with Conscious Representations via Fibre Bundles

If the anatomical separation between the executive processes themselves is fuzzy at the moment, the anatomical separation between these executive processes and the representations they operate on appears to be rather clear-cut. Representations embodied in posterior cortical areas, in particular multimodal areas in the parietal and temporal lobes (especially a region in the posterior superior temporal sulcus), interact with executive processes by way of two-way connections. The temporal and parietal lobes are extensively interconnected with the executive processes in the prefrontal lobes by several different white matter fibre tracts, called association fibres (Schmahmann and Pandya, 2006). These bundles of fibres, also known as fasciculi, are made up of millions of connecting fibres, which are axons protected by an insulating myelin sheath. The fibre pathways are reciprocal. “Thus, these pathways provide particular prefrontal areas with sensory-specific or multimodal information and, at the same time, provide the means by which prefrontal cortical areas can regulate information processing in the posterior cortical areas” (Petrides and Pandya, 2002, p45).

These views of executive processes also combine well with some of the emerging theories of consciousness. Baars has developed a cognitive theory of consciousness, according to which consciousness is seen as a global broadcasting system, which different input processors compete for access to. Representations are held in consciousness so that they can be further processed by any of a number of modules. Despite the widely (among philosophers at least) disparaged Cartesian theatre, Baars is happy to use the theatre metaphor: “Consciousness in this metaphor resembles a bright spot on the stage of immediate memory, directed by a spotlight of attention under executive guidance. Only the bright spot is conscious, while the rest of the theatre is dark and unconscious” (Baars, 2005, p46). And “behind the scenes, an invisible (unconscious) director and playwright try to exercise executive control over the actor and the spotlight” (Baars *et al*., 2003, p672). Recently, some neuroscientists have suggested that something of the sort Baars posits could be accomplished by the working memory areas located in the dorsolateral prefrontal cortex, coupled with multimodal sensory integration areas in the posterior of the cortex (Dehaene and Naccache, 2001).

## Concluding Remarks [see also Figure 1]

**Figure 1:**
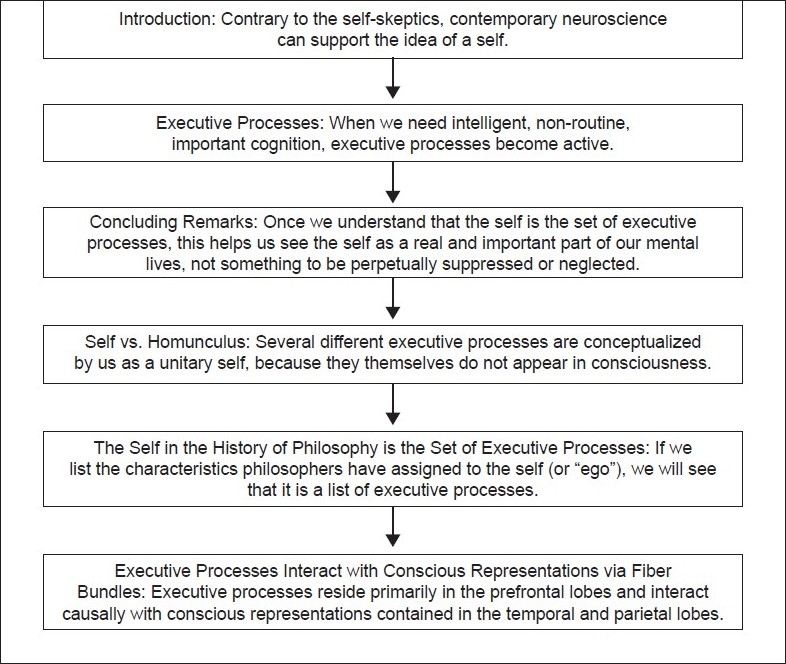
Flowchart of paper

One interesting consequence of this view is that the desire of meditators to banish the self may be therapeutic in the short term. Given the strong connections between parts of the prefrontal cortex (especially orbitomedial areas) and the autonomic system, the powerful stress-reducing effect of meditation is understandable. But a long-term desire to abolish this self, recommended by some sects of Zen Buddhism, might not be desirable, given the amount of cortex given over to it. Perhaps, instead we should cultivate efficient and stress-free ways of using our executive processes. They also need to be kept in proper proportions to one another. The inhibitory processes rage out of control in obsessive-compulsive disorder; some people so relentlessly plan for the future that they ignore the present; others annoyingly attempt to correct whatever anyone else says to them. Often the activation of executive processes brings with it a quick anger, at the sudden effort required or the jarring effect of plans thwarted. But the executive processes are a vital part of our nature, perhaps the main thing that makes us so much more flexible and adaptable than all the other animals. Dolphins and whales, for instance, despite their huge brains (with extremely large cortices, although with fewer layers than ours, typically only three or four) will beach themselves in large numbers, and when freed, swim right back to shore. Our executive processes give us the power to learn quickly after a single failure, and the power to create and solve novel problems.

### Take-home message

We can unify our philosophical and neuroscientific streams of thought by identifying the self of philosophy with the executive processes of neuroscience. Executive processes produce an active presence in consciousness that is responsible for the philosophical debate on the self. This makes sense of large portions of philosophical history, and helps us to conceptualise our current findings in neuroscience.

## Questions That This paper Raises

1. When do we identify with the workings of the executive processes and when do we not? For instance, Socrates seems to have thought that the inhibitory voice of conscience was not his own mind, but the voice of an external god, which he called a daemon.
2. Might dissociative identity disorder be conceptualised as one person having several different executive profiles, in the sense that personality can be captured by ’executive style’? Some personalities have a strong inhibitory component, for instance.
3. Can a clean separation be made between the executive processes and the representations they operate on?
4. Can mental representations be conscious without interacting with executive areas? And, can representations that are not currently conscious interact with executive areas?

## About the Author

*William Hirstein is Chair of the Philosophy Department at Elmhurst College, in Elmhurst, Illinois, USA. He received his PhD from the University of California, Davis, in 1994. His graduate and postdoctoral studies were conducted under the supervision of John Searle, V. S. Ramachandran and Patricia Churchland. He is the author of several books, including On the Churchlands (Wadsworth, 2004) and Brain Fiction: Self-Deception and the Riddle of Confabulation (MIT, 2005). His other interests include autism, sociopathy, brain laterality and the misidentification syndromes.*
